# Deficiency of the Circadian Clock Gene *Bmal1* Reduces Microglial Immunometabolism

**DOI:** 10.3389/fimmu.2020.586399

**Published:** 2020-12-08

**Authors:** Xiao-Lan Wang, Samantha E. C. Wolff, Nikita Korpel, Irina Milanova, Cristina Sandu, Patrick C. N. Rensen, Sander Kooijman, Jean-Christophe Cassel, Andries Kalsbeek, Anne-Laurence Boutillier, Chun-Xia Yi

**Affiliations:** ^1^ Université de Strasbourg, Laboratoire de Neuroscience Cognitives et Adaptatives (LNCA), Strasbourg, France; ^2^ Department of Endocrinology and Metabolism, Amsterdam University Medical Center (UMC), University of Amsterdam, Amsterdam, Netherlands; ^3^ Laboratory of Endocrinology, Amsterdam University Medical Center (UMC), University of Amsterdam, Amsterdam Gastroenterology & Metabolism, Amsterdam, Netherlands; ^4^ Netherlands Institute for Neuroscience (NIN), Royal Dutch Academy of Arts and Sciences (KNAW), Amsterdam, Netherlands; ^5^ Centre National de la Recherche Scientifique, Université de Strasbourg, Institut des Neurosciences Cellulaires et Intégratives, Strasbourg, France; ^6^ Department of Medicine, Divison of Endocrinology, and Einthoven Laboratory for Experimental Vascular Medicine, Leiden University Medical Center, Leiden, Netherlands; ^7^ CNRS UMR 7364, LNCA, Strasbourg, France

**Keywords:** inflammation, palmitic acid, cellular metabolism, oxidative stress, microglia

## Abstract

Microglia are brain immune cells responsible for immune surveillance. Microglial activation is, however, closely associated with neuroinflammation, neurodegeneration, and obesity. Therefore, it is critical that microglial immune response appropriately adapts to different stressors. The circadian clock controls the cellular process that involves the regulation of inflammation and energy hemostasis. Here, we observed a significant circadian variation in the expression of markers related to inflammation, nutrient utilization, and antioxidation in microglial cells isolated from mice. Furthermore, we found that the core clock gene-Brain and Muscle Arnt-like 1 (*Bmal1*) plays a role in regulating microglial immune function in mice and microglial BV-2 cells by using quantitative RT-PCR. *Bmal1* deficiency decreased gene expression of pro-inflammatory cytokines, increased gene expression of antioxidative and anti-inflammatory factors in microglia. These changes were also observed in *Bmal1* knock-down microglial BV-2 cells under lipopolysaccharide (LPS) and palmitic acid stimulations. Moreover, Bmal1 deficiency affected the expression of metabolic associated genes and metabolic processes, and increased phagocytic capacity in microglia. These findings suggest that *Bmal1* is a key regulator in microglial immune response and cellular metabolism.

## Introduction

Microglia serve as the brain macrophages with immune-modulating and phagocytic capabilities. Microglial activation associated neuroinflammation has been firmly linked to the development and progression of neurodegenerative diseases, such as Alzheimer’s disease, Parkinson’s disease, and Huntington’s disease (1). Severe systemic inflammation, such as sepsis, triggers microglial inflammatory activation which leads to neuronal injury and cognitive impairments in both humans (2, 3) and rodents (4). High-fat diet-induced chronic microglial inflammation results in neuronal loss and obesity (5, 6). Our previous study shows that microglial activation follows a circadian rhythm in rodents (7).

Circadian rhythms are involved in the regulation and maintenance of various physiological processes, including immune responses, energy metabolism, and memory formation (8–10). A growing body of literature shows that endogenous circadian clock function plays a crucial role in the control of many cellular processes that affect overall physiology (11–17). For example, macrophages or microglial clock gene modulates the production of cytokines, following an immune challenge (18–20). Besides the involvement of clock genes, it has also been shown that the immune activity is highly dependent on cellular metabolic processes (21–23); reduced glucose or lipid utilization inhibits microglial activation and inflammation (22, 24). However, it is still unclear whether the intrinsic clock regulates microglial immune activity through modulation of cellular metabolism.

At the molecular level, the circadian clock machinery is based on transcriptional-translational feedback loops, which are present in almost every mammalian cell (25). The transcriptional factor Bmal1 (Brain and Muscle Arnt-like 1)/Clock complex activates the expression of the period genes (*Per1*, *Per2*) and cryptochrome genes (*Cry1*, *Cry2*). Per/Cry complex suppresses its own transcription by inhibiting the activity of the Bmal1/Clock complex (25, 26). Nuclear receptor subfamily 1, group D, member 1 (Nr1d1), and RAR-related orphan receptors (Rors) fine-tune the transcription of *Bmal1*. Apart from the autoregulation, clock transcription factors also control the expression of other genes, such as the gene-D site albumin promoter binding protein (*Dbp*), by binding to their promoter (27). In the current study, we focus on the core clock gene-*Bmal1*, which is highly expressed during the light phase in microglia in mice (28).

Bmal1 is closely linked with energy metabolism (29–32), redox hemostasis (13, 33), and immune responses (19). Cellular energy metabolism and redox hemostasis regulate immune cell function, including those of microglia (13, 24). This suggests a possible link between circadian clock-*Bmal1* and the microglial immune response, as well as cellular energy metabolism and redox hemostasis. Here, we used the global Bmal1 knockout mice which show a complete loss of their circadian rhythms (34). We found a significantly reduced inflammatory and metabolic associated gene expression in microglia isolated from Bmal1 knockout mice. The decrease of inflammatory markers was also observed in *Bmal1* knocked down microglial BV-2 cells under LPS and palmitic acid stimulations.

## Materials and Methods

### Animals

Mice were housed in temperature (22 ± 1°C) and humidity (55 ± 5%) controlled room under a 12h/12h light/dark cycle {[lights on at 07:00 h, zeitgeber time 0 (ZT0)]}, with free access to food and water. B6.129-*Arntl^tm1Bra^*/J mice (34) (Jax stock #009100) were obtained from the Jackson Laboratory. The helix-loop-helix domain within exon 4 and all of exon 5 were replaced to create the mutation. B6.129-*Arntl^tm1Bra^*/J mice have a C57BL/6J background. The following primers were used for genotyping: common: 5’-GCC CAC AGT CAG ATT GAA AAG-3’; wild type reverse: 5’- CCC ACA TCA GCT CAT TAA CAA-3’; mutant reverse: 5’- GCC TGA AGA ACG AGA TCA GC-3’. Mutant band: 162 bp; wild type band: 329 bp; heterozygote band: 162 bp and 329 bp. The mice containing only a mutant band were regarded as Bmal1 knockout (*Bmal1* KO). C57BL/6J mice served as control (Ctrl) (**Supplementary Figure 1**). *Bmal1* KO male mice were killed at ZT6 at the age of 3 months. Experimental protocol (NIN18.30.02) and animal care complied with the institutional guidelines of the Netherlands (Amsterdam, the Netherlands).

C57BL/6J male mice used for microglial isolation were sacrificed at 8-time points from ZT0 at 10 weeks old. This study was approved by and performed according to the guidelines of the Institutional Animal Care and Use Committee of the Netherlands (Leiden, the Netherlands).

Two transgenic mice lines (*Cx3cr1* ^CreER^ mice, Jax mice stock no: 021160; *Bmal1* ^lox/lox^ mice, Jax mice stock no: 007668) were used to generate microglia-specific Bmal1 KO mice (*Bmal1* lox-homozygous and Cre-positive) and Ctrl mice (Cre-positive, but with a *Bmal1* wild-type sequence). Experimental protocols and animal care were in compliance with institutional guidelines and international laws and policies. Our project has been reviewed and approved by the national and regional ethics committee in Strasbourg (France). Experimental protocols and animal care were in compliance with the institutional guidelines (council directive 87/848, October 19, 1987, Ministère de l’agriculture et de la Forêt, Service Vétérinaire de la Santé et de la Protection Animale) and international laws (directive 2010/63/UE, February 13, 2013, European Community) and policies. Our project has been reviewed and approved by the French national and regional ethics committee (APAFIS#6822-2016092118336690v3).

### Acute Isolation of Microglia From Adult Mice Brain Tissue

Mice were decapitated, and brains were homogenized in RPMI medium (21875-034, Gibco) with a 15 ml Dounce homogenizer on ice until the sample is fully homogeneous, without any visible tissue fragments. The final homogenate was filtered through a 70 μm cell strainer (431751, Corning). Following 5 min centrifugation at 380 g at 4°C, cell pellets were resuspended with 7 ml RPMI medium (11875093, Gibco) and mixed with 3 ml stock isotonic Percoll (SIP) solution which was made by mixing one part 10x HBSS (14185052, Gibco) in nine parts of Percoll plus (GE17-5445-01, Sigma-Aldrich). The cell suspension was then layered slowly on top of 2 ml of 70% Percoll solution which was prepared by mixing three parts of HBSS (14170112, Gibco) with seven parts SIP in a new 15 ml falcon and centrifuged at 500 g speed for 30 min at 18°C, with minimal acceleration and break rate. After centrifugation, the fuse interphases were transferred into a new 15 ml falcon with 8 ml HBSS and centrifuged at 500 g for 7 min again. The supernatant and cell debris were discarded and microglial cells were collected for RNA isolation.

### RNA Isolation From Microglia and Quantitative PCR

Total RNA was isolated from acutely isolated microglia using RNeasy Micro Kit (74004, QIAGEN) following the manufacturer’s recommendations. We used 180 ng RNA to make cDNA with a Transcriptor First Strand cDNA Synthesis Kit (04897030001, Roche) following the manufacturer’s recommendations. 4.5 ng cDNA was used to perform qPCR with SensiFAST™ SYBR^®^ No-ROX Kit (BIO-98020, Roche Bioline). The genes *Bmal1*, *Clock*, *Cry1*, *Cry2*, *Per1*, *Per2*, *Nr1d1*, *Dbp*, *Il1b*, *Tnfa*, *Il6*, *Il10*, *Nox2*, *Gsr*, *Hmox1*, *Glut5*, *Glut1*, *Lpl*, *Gls*, and *Pcx* were evaluated. Primer sequences are presented in **Supplementary Table 1**. Data were analyzed by LC480 Conversion and LinRegPCR software and normalized to the housekeeping gene hypoxanthine phosphoribosyltransferase 1 (*Hprt1*).

### Microglial BV-2 Cell Culture and Transfection

Murine microglial BV-2 cells (35) were kindly provided by Noam Zelcer (36) and cultured in Dulbecco’s Modified Eagle’s medium (DMEM, 41965-039, Gibco) supplemented with 10% fetal bovine serum (FBS, Gibco) and 100 μg/ml penicillin-streptomycin at 37°C in a humidified atmosphere containing 5% CO_2_. Microglial BV-2 cells were transfected with the *Bmal1* siRNA or scrambled siRNA (Dharmacon) by using Viromer (VB-01LB-01, lipocalyx, Germany) according to the manufacturer’s protocol. The medium was replaced 6 h after transfection and cells were incubated as usual. 24 h after transfection, cells were synchronized with 100 nM of dexamethasone (Sigma-Aldrich) for 2 h, then washed with PBS, and followed by exposure to lipopolysaccharide (LPS, E. coli O111:B4, 100 ng/ml, L4391, Sigma-Aldrich), palmitic acid (P0500, Sigma-Aldrich) or vehicle, and finally harvested at the appropriate time points. The time at which cells were washed with PBS was defined as 0.

### Palmitic Acid and Bovine Serum Albumin Conjugation Protocol

Palmitic acid was dissolved in 150 mM NaCl with robust shaking at 70°C. Fatty acid-free BSA (A8806, Sigma-Aldrich) was dissolved in 150 mM NaCl at 37°C to make a 3.2 mM BSA solution. Half of the BSA solution was mixed with the same volume of palmitic acid solution at 37°C while stirring overnight to make palmitic acid-BSA conjugated solution (5:1 molar ratio palmitic acid: BSA). Half of the BSA solution was added to the same volume of 150 mM NaCl at 37°C while stirring overnight to make a vehicle control solution. pH was adjusted to 7.4 and solutions were filtered using a 0.22 μm syringe filter.

### RNA Isolation From BV-2 Cells

Total RNA was extracted using the High Pure RNA isolation kit (1182866500, Roche) following the manufacturer’s instructions. Complementary DNA was obtained by reverse transcription of 400 ng of total mRNA using the Transcriptor First Strand cDNA Synthesis Kit (04897030001, Roche) following the manufacturer’s recommendations.

### Primary Microglial Culture

Microglial cultures were prepared as described previously (24). Briefly, brain tissues were harvested from microglia-specific Bmal1 KO and littermate Ctrl mice at postnatal days 1–4 (P1–P4), the meninges and blood vessels were removed, and the parenchyma minced and triturated in DMEM/F12 (10565018, Gibco), containing 10% FBS, 100 μg/ml penicillin-streptomycin. Suspended cells were filtered (70 μm) and seeded on poly-L-lysine-coated flasks. Six to 10 days later, the flasks were shaken (200 rpm) for 1 h to specifically detach microglia. Microglial cells were treated with 5 μM of 4-hydroxytamoxifen for 48 h to induce Cre-LoxP recombination and excise *Bmal1*. Cells isolated from microglia-specific Bmal1 KO and Ctrl mice served as Bmal1 KO and Ctrl, respectively. Next, microglia were synchronized with 100 nM dexamethasone for 2 h, washed with PBS, and then treated with 100 ng/ml LPS for 1 h or 100 µM palmitic acid for 4 h, for final analysis.

### Western Blot Analyses

Primary microglial cells were homogenized in Laemmli buffer and sonicated for 10 s twice (ultrasonic processor, power 40%) followed by heating at 70°C for 10 min and then 100°C for 5 min. Lysates were centrifuged at 14,000 *g* for 5 min, and the supernatant was used for Western blot analyses. Proteins were loaded on Midi‐PROTEAN TGX Stain‐Free™ Precast Gels (4%–20%, Bio‐Rad) and electrotransferred onto a nitrocellulose membrane. Primary antibodies used for Western blots were rabbit anti-Bmal1 (1:500, NB100-2288, Novus Biologicals) and rabbit anti-Actin (1:2000, A2066, Sigma-Aldrich), followed by horseradish peroxidase-conjugated secondary antibodies against rabbit (1:5000, Jackson ImmunoResearch). Immunoreactive bands were detected with ECL (Clarity, Bio‐Rad) with a ChemiDoc Touch system (Bio‐Rad).

### 2-NBDG Glucose Uptake Assay

Microglial cells were plated in 96-well black plates with 3.5 x 10^4^ cells/well. After synchronization, cells were cultured in glucose-free medium with LPS or palmitic acid stimulation. At the end of treatment, 2-NBDG (ab235976, Abcam), a fluorescently-labeled deoxyglucose analog, was added to a final concentration of 200 µg/ml, and fluorescent signals were recorded at 2, 5, and 10 min by microplate reader at excitation/emission wavelengths = 480/530 nm.

### Free Fatty Acid Uptake Assay

Microglial cells were seeded in 96-well black plates with 3.5 x 10^4^ cells/well. After synchronization, cells were treated with LPS or palmitic acid in 100 µl FBS free culture medium. At the end of treatment, 100 µl fatty acid dye (TF2-C12)-loading solution (ab176768, Abcam) was incubated with microglia for 1 h at 37°C. Fluorescent signals were detected by microplate reader at 30, 45, and 60 min at excitation/emission wavelengths = 480/530 nm.

### DCFDA-Cellular Reactive Oxygen Species Detection Assay

Microglial cells were plated in 96-well black plates with 3.5 x 10^4^ cells/well. After synchronization, cells were stained by 25 µm DCFDA solution (ab113851, Abcam) for 25 min at 37°C. Fluorescent signals were detected immediately by microplate reader at excitation/emission wavelengths = 480/530 nm. Following the basal measurement, cells were challenged with 2% H_2_O_2_, and fluorescent signals were recorded up to 10 min.

### Microsphere Uptake Assay

Microspheres (17154-10, polysciences) were coated with 10% FBS at 37°C for 1 h, followed by centrifugation (12,000 rpm, 2 min) and resuspended in PBS. Coated microspheres were added to the BV-2 cells (1000 microspheres per cell) at time 0, and 1 h later, cells were washed with PBS 3 times, then fixed by 4% paraformaldehyde for 5 min, followed by PBS washing. Mounting medium with DAPI was added for confocal imaging (Leica TCS SP5; Leica, Heidelberg, Germany). Five-μm z-stack confocal images were acquired at 0.3-μm intervals, with 40×/1.3 oil objective at 1× zoom. Images were analyzed by Imaris (Bitplane AG) to measure the total volume of microspheres in every view.

### Statistical Analysis

Statistical analyses were performed using two-tailed unpaired *t*-test, one-way ANOVA, and two-way ANOVA with GraphPad Prism 8 (San Diego, California, USA). Daily variation in gene expression in microglia isolated from C57BL/6J mice was evaluated by one-way ANOVA (**Supplementary Table 2**). The daily rhythm of genes in microglia isolated from C57BL/6J mice and BV-2 cells was assessed by cosinor analysis with SigmaPlot 14.0 software (SPSS Inc, Chicago, IL, USA). Data were fitted to the following regression: y = A + B·cos(2π(x−C)/24); A is the mean level; B is the amplitude and C is the acrophase of the fitted rhythm (37). An overall *p* value (main *p* value, *P*m) was considered to indicate the rhythmicity in **Supplementary Table 3**. All data are presented as mean ± s.e.m and significance was considered at *P* < 0.05.

## Results

### Microglial Inflammatory Cytokine Genes Are Higher Expressed During the Light Phase

**Figure 1:**
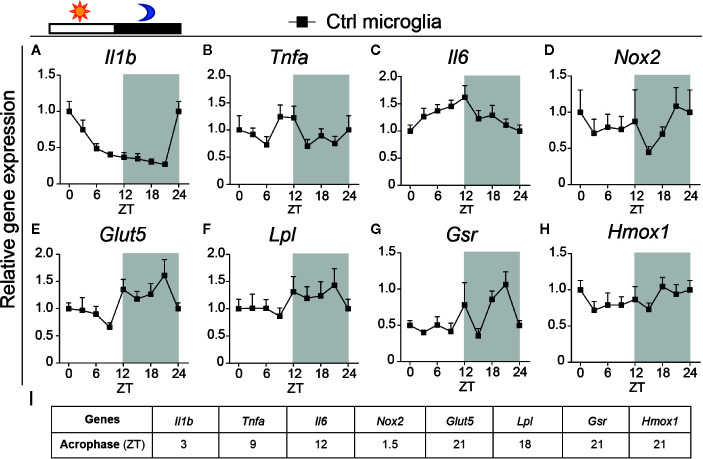
Rhythmic gene expression of inflammatory cytokines, nutrient utilization, and antioxidant anti-inflammation in microglia. **(A–H)** Relative expression of inflammatory genes interleukin 1 beta (*Il1b*) **(A)**, tumor necrosis factor (*Tnfa*) **(B)**, interleukin 6 (*Il6*) **(C)**, and oxidation and inflammation-related gene NADPH oxidase 2 (*Nox2*) **(D)**, and nutrient utilization genes facilitated glucose transporter member 5 (*Glut5*) **(E)**, lipoprotein lipase (*Lpl*) **(F)**, as well as antioxidant anti-inflammation genes glutathione reductase (*Gsr*) **(G)**, heme oxygenase 1 (*Hmox1*) **(H)**, were evaluated in isolated microglia from C57BL/6J male mice brain every 3 h (n = 6–8 samples per group per time point). ZT0 = lights on; ZT12 = lights off. Data of ZT0 and ZT24 were from the same samples. **(I)** Acrophase determined for each of the genes. Statistical significance of rhythmic expression was determined by the Cosinor analysis and one-way ANOVA. Data are presented as means ± s.e.m.

A previous rat study has demonstrated that microglial intrinsic clock genes and inflammatory cytokine genes show daily expression rhythms in the hippocampus and microglial inflammatory cytokine genes highly expressed during the light phase (38). It has also been shown that LPS treated rodents show increased sickness behavior or proinflammatory response during the light phase compared to the dark phase (38, 39). In the current mouse study, we found that in microglial cells, the gene expression of pro-inflammatory cytokines-interleukin 1 beta (*Il1b*) and interleukin 6 (*Il6*), but not tumor necrosis factor (*Tnfa*), followed a daily expression rhythm (**Figures 1A–C**, and **Supplementary Tables 2 and 3**). Both *Il1b* and *Il6* showed higher gene expression during the light phase than in the dark phase. The peak in *Tnfa* expression was also found during the light phase (**Figures 1A–C, I**). Moreover, the oxidation and inflammation-related gene NADPH oxidase 2 (*Nox2*) also showed rhythmic expression in microglia (**Figure 1D** and **Supplementary Table 3**). Together these findings suggest that microglia may have a higher innate immune activity during the light phase in mice.

### Microglial Nutrient Utilization and the Antioxidation Transcripts Show Daily Rhythmicity

In physiological conditions, microglia are highly dynamic to clean the microenvironment and maintain neuronal survival and function (40). The previous study showed that microglial activity is higher during the dark phase when mice are more active as compared with the light phase when mice are mainly resting (7). Microglial activity is also highly dependent on cellular metabolism (24). Therefore, we evaluated gene expression of facilitated glucose transporter member 5 (*Glut5*), which is highly expressed by microglia, and lipoprotein lipase (*Lpl*), which mediates lipoprotein triglyceride-derived fatty acid uptake. Both *Glut5* and *Lpl* exhibited an increased expression during the dark phase (**Figures 1E, F, I**), which suggests an increased nutrient utilization when microglia are more active. Microglial activation leads to the production of more metabolites and ROS (41, 42) that need to be eliminated to maintain proper microglial function (43–45). Thus, we checked the gene expression of glutathione reductase (*Gsr*), a key enzyme for the production of sulfhydryl form glutathione (GSH). GSH acts as a scavenger and plays a critical role in preventing oxidative stress in cells. We also evaluated the rhythmic gene expression of heme oxygenase 1 (*Hmox1*), which has antioxidant anti-inflammation properties *via* the production of carbon monoxide (CO) (44). We observed an increased expression of *Gsr* and *Hmox1* during the dark phase (**Figures 1G–I**). The gene expression of *Glut5* and *Gsr* showed significant daily variation (**Supplementary Table 2**). These data indicate that the expression of nutrient utilization and the antioxidation associated genes in microglial cells follows a daily rhythm, which is in line with their activity.

### *Bmal1* Knockout Microglia Show Decreased Gene Expression of Inflammation and Nutrient Utilization

**Figure 2:**
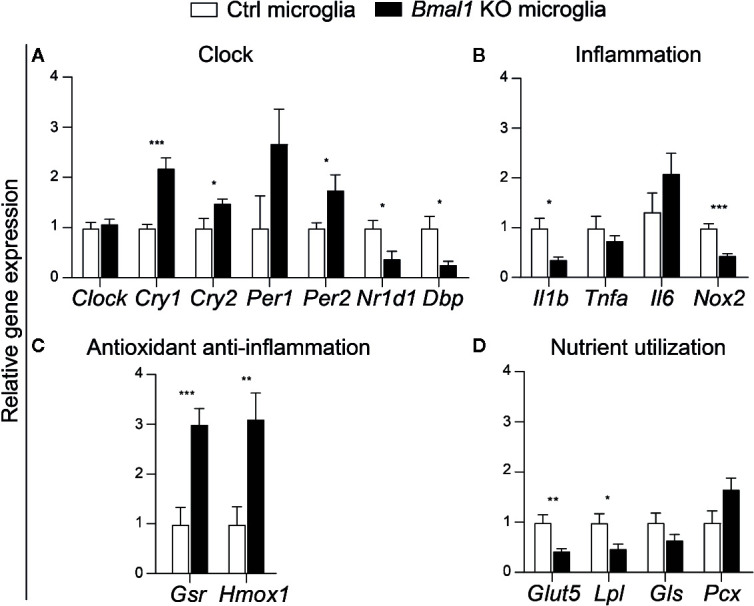
*Bmal1* KO microglia show decreased inflammation and nutrient utilization in mice. **(A–D)** Relative expression of clock genes-*Clock*, cryptochrome (*Cry1*, *Cry2*), period (*Per1*, *Per2*), Nuclear receptor subfamily 1, group D, member 1 (Nr1d1), D site albumin promoter binding protein (*Dbp*) **(A)**, inflammation-related genes *Il1b*, *Tnfa*, *Il6*, *Nox2* **(B)**, and antioxidant anti-inflammation genes *Gsr*, *Hmox1* **(C)**, as well as cellular metabolic-related genes *Glut5*, *Lpl*, glutaminase (*Gls*), and pyruvate carboxylase (*Pcx*) **(D)**, were evaluated in isolated microglia from *Bmal1* KO mice and C57BL/6J mice brain at ZT6 (n = 8 samples per group). Data were analyzed with *t*-tests and are presented as means ± s.e.m. * *P* < 0.05, ** *P* < 0.01, and *** *P* < 0.001.

The circadian clock system is associated with the innate immune activity (16). Therefore, we were interested in whether the *Bmal1* regulates microglial immunometabolism and evaluated the related gene expression in microglia isolated from *Bmal1* KO mice and controls in the middle of the light phase (ZT6). Expression of clock genes in *Bmal1* KO microglia was disturbed, with a significant increase in *Cry1*, *Cry2*, and *Per2*, as well as a decrease in *Nr1d1*, and *Dbp* (**Figure 2A**). *Il1b* and *Nox2* were significantly lower in *Bmal1* KO microglia, while *Tnfa* and *Il6* did not differ between both groups (**Figure 2B**). Moreover, *Gsr* and *Hmox1* expression were strikingly increased (**Figure 2C**). Taken together, these data suggest that *Bmal1* KO decreases inflammation and increases the anti-inflammation antioxidative effect in microglia. Furthermore, microglial *Glut5* and *Lpl* were significantly decreased in *Bmal1* KO mice (**Figure 2D**). While glutaminase (*Gls*), which is involved in glutamate utilization, and pyruvate carboxylase (*Pcx*), which participates in gluconeogenesis and lipogenesis, did not differ between the two groups (**Figure 2D**). These findings suggest reduced nutrient utilization in *Bmal1* KO microglia as compared to controls in physiological conditions.

### *Bmal1* Deletion Disturbs the Expression of Clock Genes in Microglial BV-2 Cells

**Figure 3:**
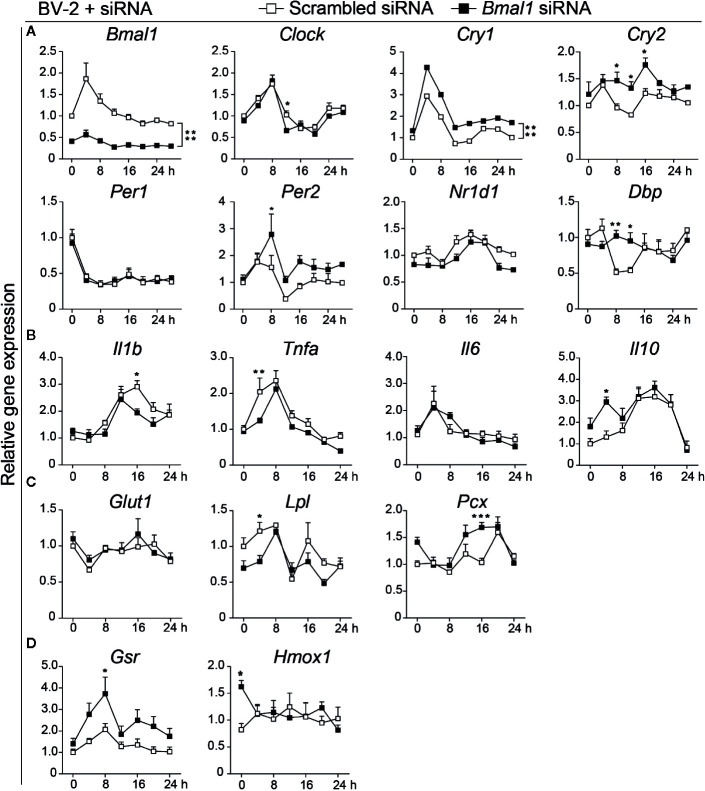
*Bmal1* deficiency decreases inflammation and nutrient utilization of microglial BV-2 cells. **(A–D)** Relative gene expression in scrambled siRNA and *Bmal1* siRNA groups (n = 3–6 samples per group per time point). Clock genes *Bmal1*, *Clock*, *Cry1*, *Cry2*, *Per1*, *Per2*, *Nr1d1*, *Dbp* **(A)**, inflammatory cytokine genes *Il1b*, *Tnfa*, *Il6*, *Il10* **(B)**, glucose and fatty acid metabolism genes *Glut1*, *Lpl*, *Pcx* **(C)**, and antioxidant genes *Gsr*, *Hmox1* **(D)** were evaluated every 4 h for 28 h after BV-2 cells were exposed to Dex. Data were analyzed with two-way ANOVA. Statistical significance of rhythmic expression was determined by Cosinor analysis. Data are presented as means ± s.e.m. * *P* < 0.05, ** *P* < 0.01, and *** *P* < 0.001.

To further study the relationships among the intrinsic clock, immune activity, cellular metabolism, and antioxidative effect, specifically in microglia, we performed experiments in microglial BV-2 cells. Rhythmic expression of clock genes can be achieved in microglial BV-2 cells after synchronization with 100 nM dexamethasone for 2 h (19). Using this cell model, we found that the inflammatory cytokines *Il1b*, *Tnfa*, interleukin 10 (*Il10*), and *Il6* showed a significant rhythmic expression after synchronization (**Supplementary Figure 2** and **Supplementary Table 3**). To evaluate whether the intrinsic clock regulates microglial function, we knocked down *Bmal1* in BV-2 cells. First, we checked the clock gene expression every 4 h for 28 h after synchronization. We observed that *Bmal1* gene expression was significantly decreased in the *Bmal1* knock-down group (*Bmal1* siRNA) compared with the control group (scrambled siRNA) (**Figure 3A**). *Bmal1* controlled genes, such as *Cry1*, *Cry2*, and *Per2*, and the *Bmal1* targeted gene *Dbp* showed a significant increase in the *Bmal1* knock-down group. While *Clock* was decreased 12 h after synchronization; *Per1* and *Nr1d1* expression did not change (**Figure 3A**). The rhythmic expression of *Bmal1* was disturbed in the *Bmal1* knock-down group, but *Clock*, *Cry1*, *Per1*, *Per2*, *Nr1d1*, and *Dbp* still showed rhythmic expression (**Supplementary Table 3**). These results indicate that *Bmal1* deletion disturbs the core clock machinery in BV-2 cells.

### *Bmal1* Deficiency Decreases the Expression of Inflammation and Nutrient Utilization Associated Genes in BV-2 Cells

To assess whether *Bmal1* deficiency also affects immune activity in microglial BV-2 cells, we evaluated the expression of inflammatory cytokine genes and related genes. As expected, expression of the pro-inflammatory cytokines *Il1b* and *Tnfa* was decreased and expression of the anti-inflammatory cytokine *Il10* was increased in the *Bmal1* knock-down group; *Il6* was not different between the two groups (**Figure 3B**). Gene expression of the glucose transporter 1 (*Glut1*), which is highly expressed in BV-2 cells, did not differ between the two groups (**Figure 3C**). While *Lpl* expression was significantly reduced and *Pcx*, which plays a crucial role in gluconeogenesis and lipogenesis, was increased in the *Bmal1* knock-down group (**Figure 3C**). These findings suggest reduced inflammation and nutrient utilization in the *Bmal1* knock-down group. Additionally, *Gsr* and *Hmox1* showed higher expression in the *Bmal1* knock-down group (**Figure 3D**). Inflammatory cytokines showed rhythmic expression in both groups (**Supplementary Table 3**). All of these data suggest that in basal conditions, *Bmal1* knock-down reduces the transcription of inflammation and nutrient utilization-related genes in BV-2 cells.

### *Bmal1* Deficient BV-2 Cells Show Less Inflammatory Gene Expression Under LPS Stimulation

**Figure 4:**
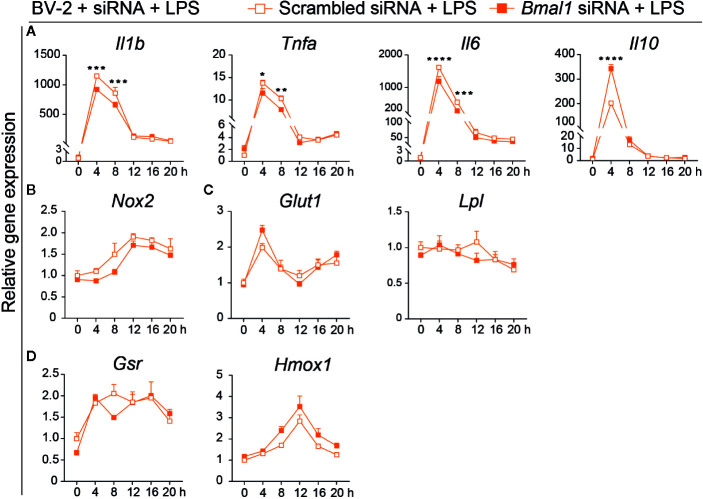
*Bmal1* knock-down reduces the pro-inflammatory gene expression and increases the antioxidative anti-inflammatory gene expression of BV-2 cells after LPS stimulation. **(A–D)** Gene expression in scrambled siRNA and *Bmal1* siRNA groups every 4 h for 20 h in the presence of LPS (n = 3–6 samples per group per time point). Pro-inflammatory cytokine genes *Il1b*, *Tnfa*, *Il6* **(A)**, anti-inflammatory cytokine gene *Il10* **(A)**, oxidative stress gene *Nox2* **(B)**, glucose metabolism gene *Glut1*, and lipid metabolism gene *Lpl* **(C)**, and antioxidant anti-inflammation gene *Gsr*, *Hmox1* **(D)** were evaluated at 6-time points after LPS exposure. Statistical significance was determined using two-way ANOVA. Statistical significance of the rhythmic expression was determined by Cosinor analysis. Data are presented as means ± s.e.m. * *P* < 0.05, ** *P* < 0.01, and *** *P* < 0.001.

To further verify the effect of *Bmal1* on the microglial immune response, we challenged the *Bmal1* knock-down and control BV-2 cells with LPS after synchronization. LPS treatment did not change the rhythmic expression of the clock genes-*Bmal1*, *Clock*, or *Per1* in either group (**Supplementary Figure 3** and **Supplementary Table 3**). The *Bmal1* knock-down group showed significantly less pro-inflammatory *Il1b*, *Tnfa*, and *Il6* expression at 4 h and 8 h after LPS treatment, and higher anti-inflammatory *Il10* expression at 4 h than the control group (**Figure 4A**). In addition to the production of inflammatory cytokines, LPS stimulation also upregulates Nox2 expression that contributes to oxidative stress (46). But there was no genotype difference in *Nox2* expression (**Figure 4B**). *Lpl*, which showed less expression in *Bmal1* deficient group in basal condition, was no difference between the *Bmal1* deficient group and controls after LPS treatment (**Figure 4C**). *Glut1*, *Gsr*, and *Hmox1* expression were similar between the two groups (**Figures 4C, D**). Taken together, these data indicate that *Bmal1* knock-down alters inflammation and nutrient utilization transcripts in BV-2 cells after LPS treatment.

### *Bmal1* Knock-Down Partially Alters the Expression of Inflammation-Related Genes in Palmitic Acid-Treated BV-2 Cells

It has been shown that consumption of a high-fat diet, especially of its main ingredient the saturated fatty acids, results in hypothalamic microglial activation and inflammation (24). It has also been demonstrated that the saturated fatty acid palmitic acid increases inflammation and oxidative stress in cultured microglial cells (47, 48). To test whether palmitic acid still induces inflammation after synchronization, cells were treated with two concentrations of palmitic acid (100 and 200 µM) for 12 h. We observed that both concentrations significantly increased *Il1b* and *Tnfa* expression; while only 100 µM palmitic acid stimulation increased *Il6* expression compared with vehicle (**Supplementary Figure 4**).

Since our previous study had shown that an HFD disturbs the expression of microglial clock genes and the daily rhythmicity in rats (49), we first studied clock genes expression in 100 µM palmitic acid-treated BV-2 cells under control and *Bmal1* knock-down conditions. Here, we observed that palmitic acid abolished the rhythmic expression of *Bmal1* and *Clock* in both groups and shifted the rhythmic expression of *Cry1*, *Cry2*, *Per2*, *Nr1d1*, and *Dbp* (**Supplementary Figure 5** and **Supplementary Table 3**). After palmitic acid treatment, clock genes showed a significant decrease at 4 h compared with 0 h. *Bmal1* expression was still lower in the *Bmal1* knock-down group than in controls from 8 h to 24 h. *Cry1*, *Cry2*, *Per2*, and *Dbp* kept an increased expression in the *Bmal1* knock-down group at some time points. While *Clock*, *Per1*, and *Nr1d1* did not differ between the two groups (**Supplementary Figure 5**).

**Figure 5:**
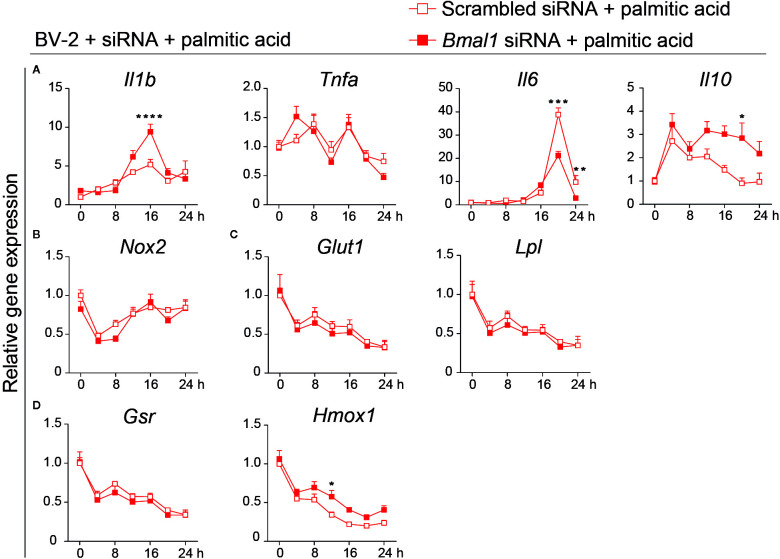
*Bmal1* knock-down partially decreases the pro-inflammatory gene expression and increases the antioxidative anti-inflammatory gene expression in palmitic acid-treated BV-2 cells. **(A–D)** Gene expression in scrambled siRNA and *Bmal1* siRNA groups every 4 h for 24 h in the presence of palmitic acid (n = 3–6 samples per group per time point). Pro-inflammatory cytokine genes *Il1b*, *Tnfa*, *Il6* **(A)**, anti-inflammatory cytokine gene *Il10* **(A)**, oxidative stress gene *Nox2* **(B)**, glucose metabolism gene *Glut1*, and lipid metabolism gene *Lpl* **(C)**, and antioxidant anti-inflammation gene *Gsr*, *Hmox1* **(D)** were evaluated at 7-time points after palmitic acid treatment. Statistical significance was determined using two-way ANOVA. Statistical significance of rhythmic expression was determined by Cosinor analysis. Data are presented as means ± s.e.m. * *P* < 0.05, ** *P* < 0.01, and *** *P* < 0.001.

Next, we evaluated whether *Bmal1* deficiency protects microglia from palmitic acid-induced inflammation. Surprisingly, *Il1b* was significantly increased at 16 h after palmitic acid treatment in the *Bmal1* knock-down group; *Il6* decreased at a later phase (20, 24 h) and *Il10* was increased at 20 h in the *Bmal1* knock-down group (**Figure 5A**). *Tnfa* expression did not differ between the control and *Bmal1* knock-down groups (**Figure 5A**). A previous study has shown that palmitic acid treatment induces oxidative stress through Nox2 upregulation, which also plays an important role in inflammation (50). Here the gene expression of *Nox2*, *Glut1*, *Lpl*, and *Gsr* showed no differences between the two groups (**Figures 5B–D**). But *Bmal1* deficiency increased *Hmox1* expression at 12 h after palmitic acid stimulation (**Figure 5D**). Together, these data suggest that *Bmal1* knock-down totally disturbs the rhythmic expression of clock genes, and only partially reduces the expression of palmitic acid-induced inflammation and oxidative stress associated genes in BV-2 cells.

### Bmal1 Deficiency Alters Metabolic Processes and Increases Phagocytosis of Microglia

**Figure 6:**
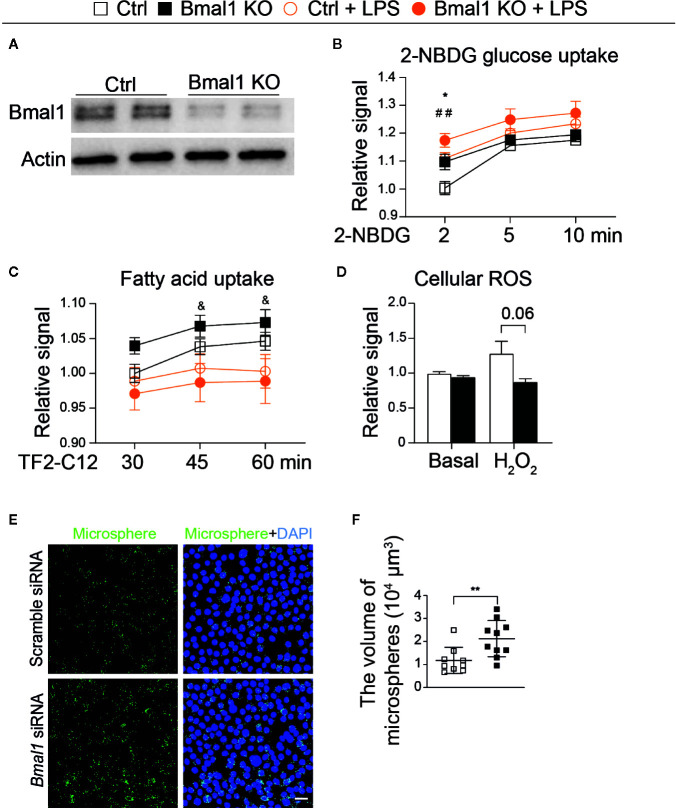
Knockout of Bmal1 affects energy utilization of microglial cells. **(A)** Bmal1 KO efficiency in microglia (n = 4 samples per group). **(B, C)** 2-NBDG glucose uptake (B, n = 9–10 samples per group), and free fatty acid uptake (**C**, n = 5 samples per group) of Ctrl and Bmal1 KO microglia under basal and LPS conditions. *Bmal1 KO *vs.* Ctrl; ## Ctrl + LPS *vs* Ctrl; & Bmal1 KO + LPS *vs.* Bmal1 KO. **(D)** Cellular ROS level in Ctrl and Bmal1 KO microglia under basal and H_2_O_2_ stimulation (n = 5 samples per group). **(E)** Image of microspheres in BV-2 cells in scrambled siRNA and Bmal1 siRNA groups after 1 h incubation (n = 9-10 samples per group). Scale bar, 30 µm. **(F)** The uptake of microspheres. Statistical significance was determined using two-way ANOVA and unpaired *t*-test. Data are presented as means ± s.e.m. *P < 0.05, ^&^ *P* < 0.05, and ^##^ *P* < 0.01.

To affirm the Bmal1 effects on nutrient utilization, we evaluated the dynamic process of glucose and free fatty acid uptake in primary microglial cells. Bmal1 was significantly decreased in Bmal1 KO microglia compared with Ctrls 48 h after the 4-hydroxytamoxifen treatment (**Figure 6A**). At 2 min after the treatment of 2-NBDG, we observed an increased glucose uptake in Bmal1 KO microglia compared to Ctrls in basal condition; LPS stimulation increased glucose uptake in Ctrls, however, which was not observed in Bmal1 KO microglia when compared with the basal conditions (**Figure 6B**). No differences were observed when extending the incubation of 2-NBDG to 5 and 10 min (**Figure 6B**). Interestingly, Bmal1 KO microglia treated with LPS showed less free fatty acid uptake compared with their basal condition at 45 and 60 min, respectively (**Figure 6C**). But there was no genotype difference in free fatty acid uptake at each time point (**Figure 6C**). Moreover, we saw less cellular ROS activity in Bmal1 KO microglia than Ctrl microglia under H2O2 stimulation, while no genotype difference in basal condition (**Figure 6D**). No differences were observed in glucose and free fatty acid uptake after palmitic acid stimulation (**Supplementary Figure 6**). Surprisingly, the phagocytic capacity was significantly increased in Bmal1 knock-down BV-2 cells (**Figures 6E, F**). These data indicate that Bmal1 KO microglia shift glucose and lipid utilization, and show an increase of phagocytosis.

## Discussion

**Figure 7:**
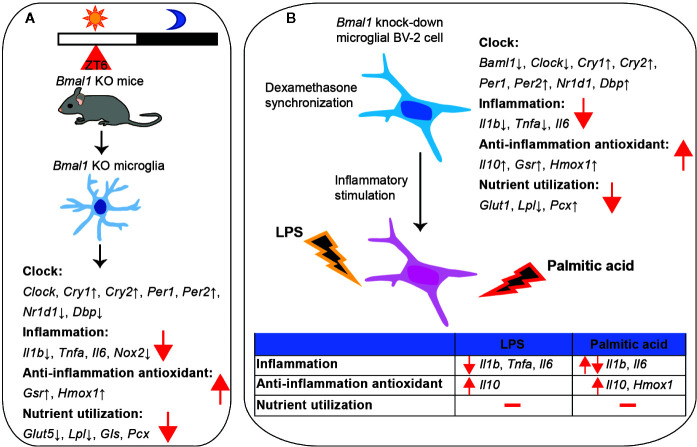
Gene expression summary. **(A)** Summary of *Bmal1* KO effects in microglia isolated from mice. **(B)** Summary of *Bmal1* knock-down effects in microgial BV-2 cells.

Circadian rhythms are closely related to immunity and metabolism (10, 51, 52). However, it was still unclear whether and if so, how the endogenous circadian clock regulates microglial immune response and metabolism. Here, we observed a significant daily rhythmic expression of inflammation, nutrient utilization, and antioxidation related genes in microglia isolated from mice. We further found that deficiency of *Bmal1* affected inflammation and nutrient utilization associated gene expression in microglial cells (**Figure 7**).

We evaluated gene expression of inflammatory cytokines in *Bmal1* knock-down microglia in both *Bmal1* KO mice and BV-2 cells. Lacking *Bmal1* decreased pro-inflammatory gene expression and increased anti-inflammatory gene expression in microglial cells. It is known that *Bmal1* expression is higher during the light phase than the dark phase (28), which is consistent with pro-inflammatory cytokine gene expression in microglia in mice under light/dark conditions. This may explain why *Bmal1* deficient microglia showed less inflammation. Moreover, Bmal1 as a transcription factor not only controls clock genes expression, but also regulates the expression of other genes *via* specific binding regulatory elements [E-box, D-box, and RORE (Ror/Rev-erb-binding element)] in their promoters (53–56). It has been shown that Bmal1 directly regulates *Il6* transactivation in microglia; conditional Bmal1 deficiency in microglial cells attenuates the ischemic neuronal damage in mice (19). In macrophages, Bmal1 regulates inflammation *via* controlling the gene expression of *Nrf2*, which plays a critical role in the innate immune system (13). Bmal1 deficiency in bone marrow-derived macrophages leads to increased IL-1β expression and increases polymicrobial infection in mice (13, 57). However, myeloid cell Bmal1 deletion protects against bacterial infection in the lung (58). We noticed that in peritoneal macrophages, *Bmal1* is highly expressed during the dark phase, which is different from the daily rhythm in microglia (57). In rat monocytes, *Bmal1* does not show a clear daily rhythm in gene expression, while *Tnfa* shows a high expression during the dark phase (49). These data suggest that Bmal1 regulates the innate immune system, but that the rhythmicity of clock genes and inflammatory cytokines is heterogeneous among innate immune cells and depends on the specific tissue.

Furthermore, we observed that *Bmal1* deletion affected the expression level of other clock genes, especially *Cry1*, *Cry2*, and *Per2* were significantly increased in *Bmal1* KO microglia. It has been shown that overexpression of Cry1 significantly decreases inflammation in atherosclerotic mice (59), whereas the absence of Cry1 and Cry2 leads to increased pro-inflammatory cytokine expression in macrophages (60). *Per2* negatively regulates the expression of pro-inflammatory cytokines in zebrafish (61). *Bmal1* deficiency-induced the increased gene expression of *Cry1*, *Cry2*, and *Per2* may also contribute to the reduced inflammation of *Bmal1* KO microglial cells.

Circadian clocks are fundamental physiological regulators in energy homeostasis and the immune system. Diurnal oscillations in glucose and lipid metabolism are due in part to daily changes in energy requirements (62). As the resident brain macrophages, microglia provide continually surveillant and scavenging functions in the brain (40). Here, we found that microglial glucose and lipid utilization transcripts show clear daily rhythmicity, and both are significantly higher during the regular activity period in mice (i.e. the dark period). *Bmal1* deficiency decreased the expression of nutrient utilization related genes in microglia under basal condition and shifted the metabolic processes under LPS stimulation. Reduced substrates utilization was observed in Bmal1 KO skeletal muscle (30, 63). A recent study showed that Bmal1 deletion also protects mice from insulin resistance induced by circadian disruption (64).

Prior work has demonstrated that ROS production and scavenging related genes exhibit a time-of-day specific expression under diurnal and circadian conditions (65). We found that *Bmal1* deficiency protected microglia from oxidative damage and inflammation. On the other hand, neuronal Bmal1 deletion causes oxidative damage and impaired expression of the redox defense gene (33), which suggests that the effect of Bmal1 on oxidative stress is different depending on the cell type involved. Moreover, microglia can polarize into a pro-inflammatory or an anti-inflammatory phenotype (66, 67). *Bmal1* KO microglia may polarize into the anti-inflammatory state by increasing IL-10 gene expression to facilitate phagocytosis of cell debris and antagonizing the pro-inflammatory response.

Recently, it has been shown that the intrinsic circadian clocks, such as REV-ERBα, REV-ERBβ, and Bmal1, affect microglial amyloid-β clearance in the 5XFAD mouse model of Alzheimer’s disease (68). Based on our findings, future work should measure inflammatory cytokine levels in combination with mitochondrial fuel utilization in microglia lacking Bmal1. Furthermore, the immune response in microglia-specific Bmal1 knockout mice still needs to be explored. Microglia are closely related to neuroinflammation, neurodegeneration, and obesity. Thus, future work should evaluate Bmal1 knockout microglial function and neuronal changes in mice under different stressful conditions, such as LPS and high-fat diet, or in combination with neurodegenerative diseases.

In conclusion, Bmal1 is a critical regulator in microglial function. Bmal1 deficiency alters microglial inflammatory profile, including inhibiting the gene expression of pro-inflammatory cytokines and elevating the expression of antioxidative anti-inflammatory factors, as well as affects microglial nutrient utilization and phagocytosis. Our work indicates that targeting the molecular biological clock-*Bmal1* in microglia might be a new approach to treat inflammation-related diseases in the brain.

## Data Availability Statement

The original contributions presented in the study are included in the article/**Supplementary Material**, further inquiries can be directed to the corresponding author/s.

## Ethics Statement

The animal study was reviewed and approved by Netherlands Institute for Neuroscience, Royal Dutch Academy of Arts and Sciences, Amsterdam, the Netherlands; Department of Medicine, Division of Endocrinology, and Einthoven Laboratory for Experimental Vascular Medicine, Leiden University Medical Center, Leiden, the Netherlands; French national and regional ethics committee in Strasbourg.

## Author Contributions

X-LW, SW, NK, IM, and SK performed the experiments. X-LW analyzed the data and wrote the manuscript. X-LW, C-XY, and A-LB designed the study. AK and A-LB edited the manuscript. All authors contributed to the article and approved the submitted version

## Funding

This work was supported by the “NeuroTime” Erasmus Mundus program, University of Strasbourg, CNRS and ANR-18-CE16-0008 (to A-LB).

## Conflict of Interest

The authors declare that the research was conducted in the absence of any commercial or financial relationships that could be construed as a potential conflict of interest.
