# Supplementary material for: Deficiency of the Circadian Clock Gene Bmal1 Reduces Microglial Immunometabolism
Source: Front Immunol. 2020 Dec 8;11:586399. doi: 10.3389/fimmu.2020.586399 (PMC7753637; doi:10.3389/fimmu.2020.586399)
Supplement: Supplementary file 1 [file DataSheet_1.docx]

**Deficiency of the circadian clock gene *Bmal1* reduces microglial immunometabolism**

**Xiao-Lan Wang^1,2,3^，Samantha E.C. Wolff^2,3^，Nikita Korpel^2,3,4^，Irina Milanova^2,3^，Cristina Sandu^5^, Patrick C.N. Rensen^5^，Sander Kooijman^5^，Jean-Christophe Cassel^1,6^，Andries Kalsbeek^2,3,4^，Anne-Laurence Boutillier^1,6^，Chun-Xia Yi^2,3.4^**

^1^Université de Strasbourg, Laboratoire de Neuroscience Cognitives et Adaptatives (LNCA), Strasbourg, France

^2^Department of Endocrinology and Metabolism, Amsterdam University Medical Center (UMC), University of Amsterdam, Amsterdam, the Netherlands

^3^Laboratory of Endocrinology, Amsterdam University Medical Center (UMC), University of Amsterdam, Amsterdam Gastroenterology & Metabolism, Amsterdam, the Netherlands

^4^Netherlands Institute for Neuroscience, an Institute of the Royal Netherlands Academy of Arts and Sciences, Amsterdam, the Netherlands

^5^Centre National de la Recherche Scientifique, Université de Strasbourg, Institut des Neurosciences Cellulaires et Intégratives, Strasbourg, France

^6^Department of Medicine, Divison of Endocrinology, and Einthoven Laboratory for Experimental Vascular Medicine, Leiden University Medical Center, Leiden, the Netherlands

^7^CNRS UMR 7364, LNCA, Strasbourg, France

**Correspondence**

Xiao-Lan Wang M.D. Ph.D.

Department of Endocrinology and Metabolism,

Amsterdam UMC, University of Amsterdam.

Meibergdreef 9, 1105AZ, Amsterdam, The Netherlands

Tel: +31 20 5664807

Fax: +31 20 6917682

E-mail: [xiaolan.wang@etu.unistra.fr](mailto:xiaolan.wang@etu.unistra.fr)

**Supplementary Figures**

**
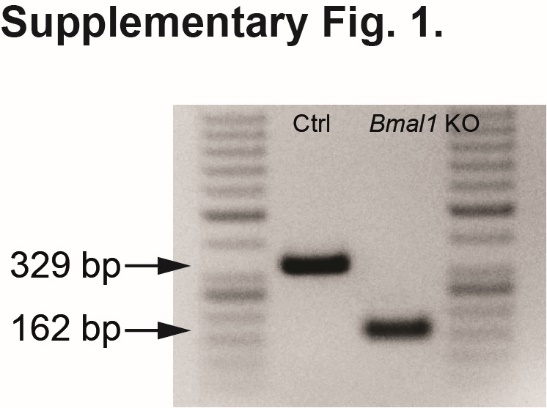
**

Genotyping results of Ctrl and *Bmal1* KO mice.

**
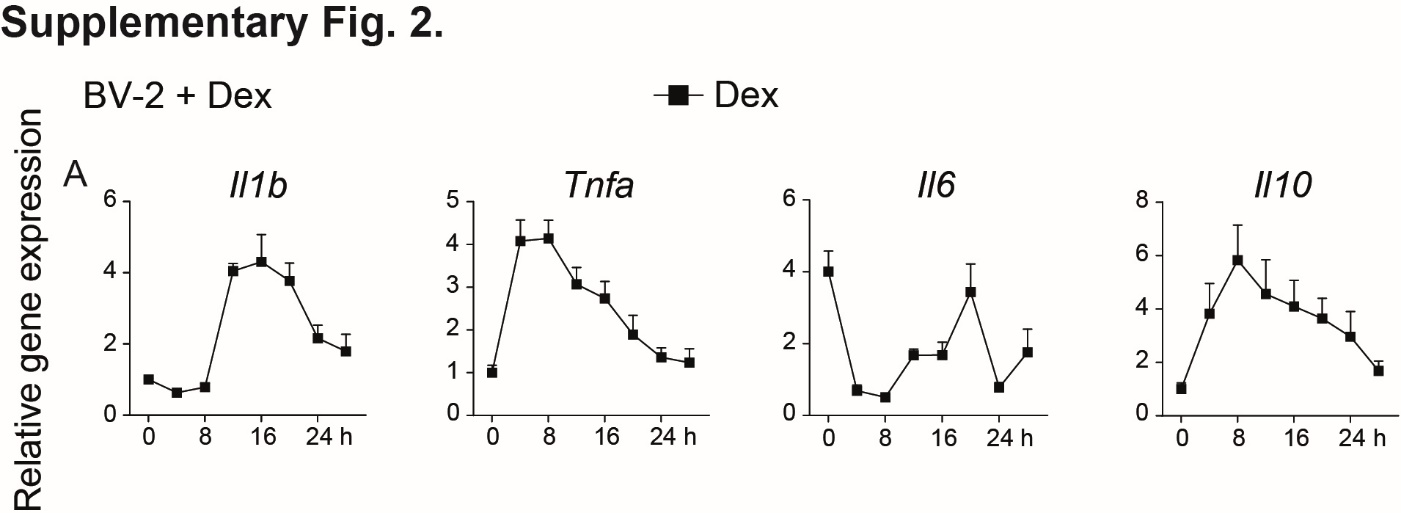
**

Rhythmic expression of inflammatory genes in microglial BV-2 cells after synchronization. (A) BV-2 cells were synchronized with dexamethasone (Dex, 100 nM) for 2 h, followed by RNA collection every 4 h for 28 h. Inflammatory genes-*Il1b*, *Tnfa*, *Il6*, *Il10* were evaluated by quantitative RT-PCR (n = 4-6 samples per group per time point). Statistical significance of rhythmic expression was determined by Cosinor analysis. Data are presented as means ± s.e.m.

**
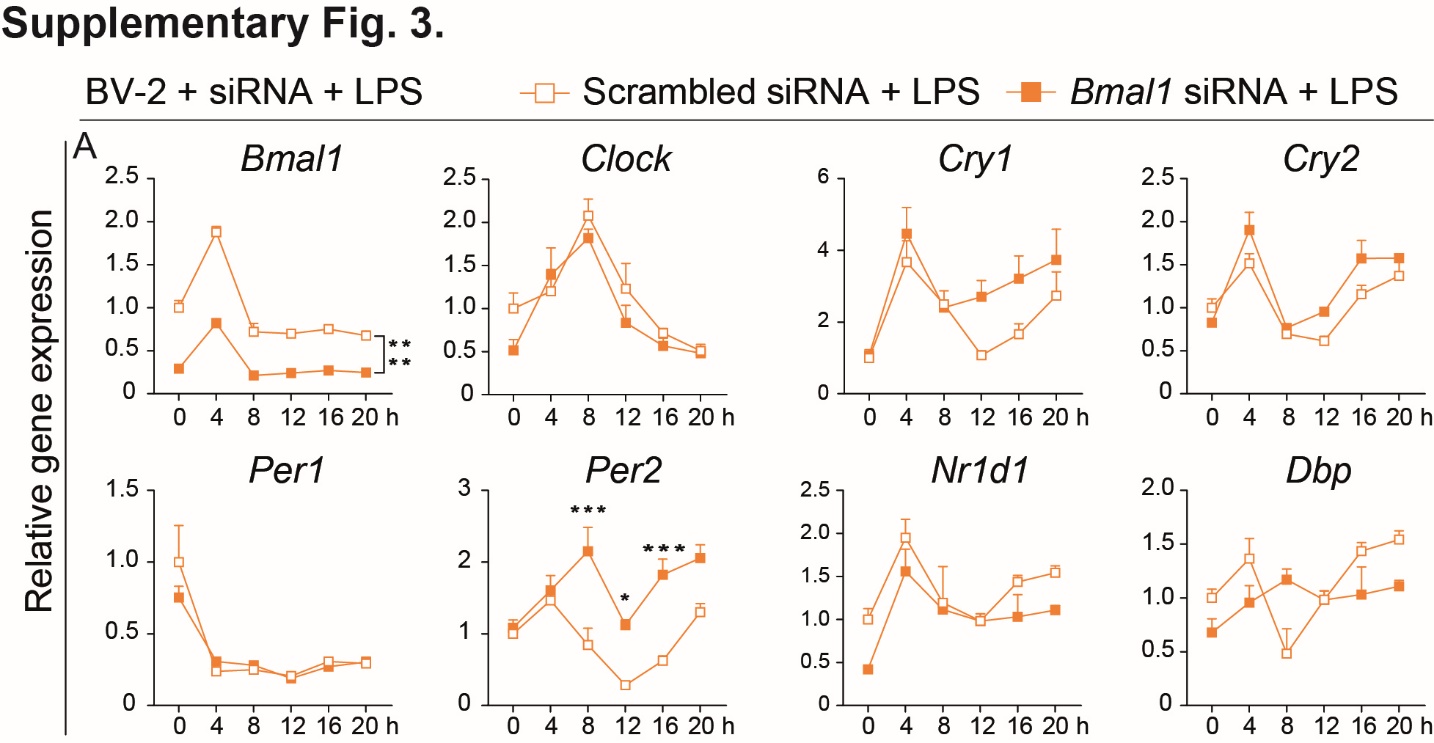
**

Clock gene expression in scrambled siRNA and *Bmal1* siRNA groups in BV-2 cells after LPS stimulation. (A) The expression of clock genes was evaluated every 4 h for 20 h in the presence of LPS (n = 3-6 samples per group per time point). Statistical significance was determined using two-way ANOVA. Statistical significance of the rhythmic expression was determined by Cosinor analysis. Data are presented as means ± s.e.m. * *P* < 0.05, ** *P* < 0.01, and *** *P* < 0.001.

**
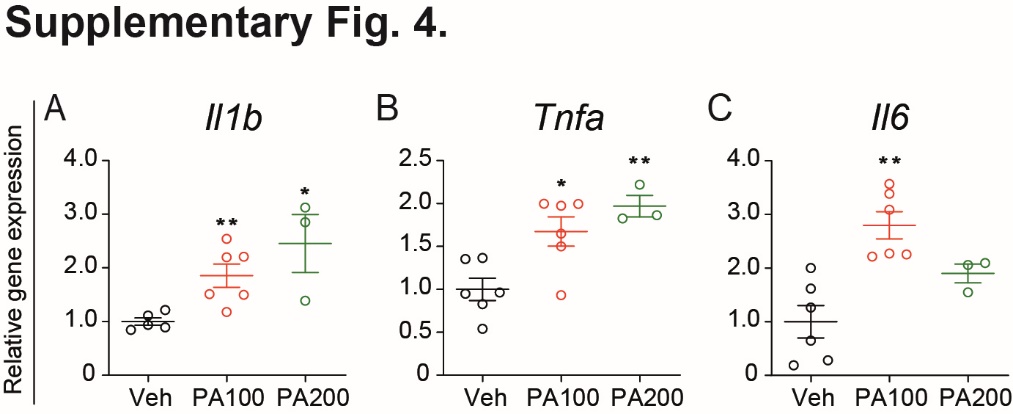
**

Palmitic acid treatment increases the pro-inflammatory cytokine gene expression in microglial BV-2 cells after synchronization. (A-C) Relative gene expression of pro-inflammatory cytokine genes *Il1b*, *Tnfa*, *Il6*, 12 h after palmitic acid treatment at different concentrations (PA100, palmitic acid 100 µM; PA200, palmitic acid 200 µM) (n = 3-6 samples per group). Statistical significance was determined using unpaired *t*-test. Data are presented as means ± s.e.m. Compared with Veh, * *P* < 0.05, and ** *P* < 0.01.


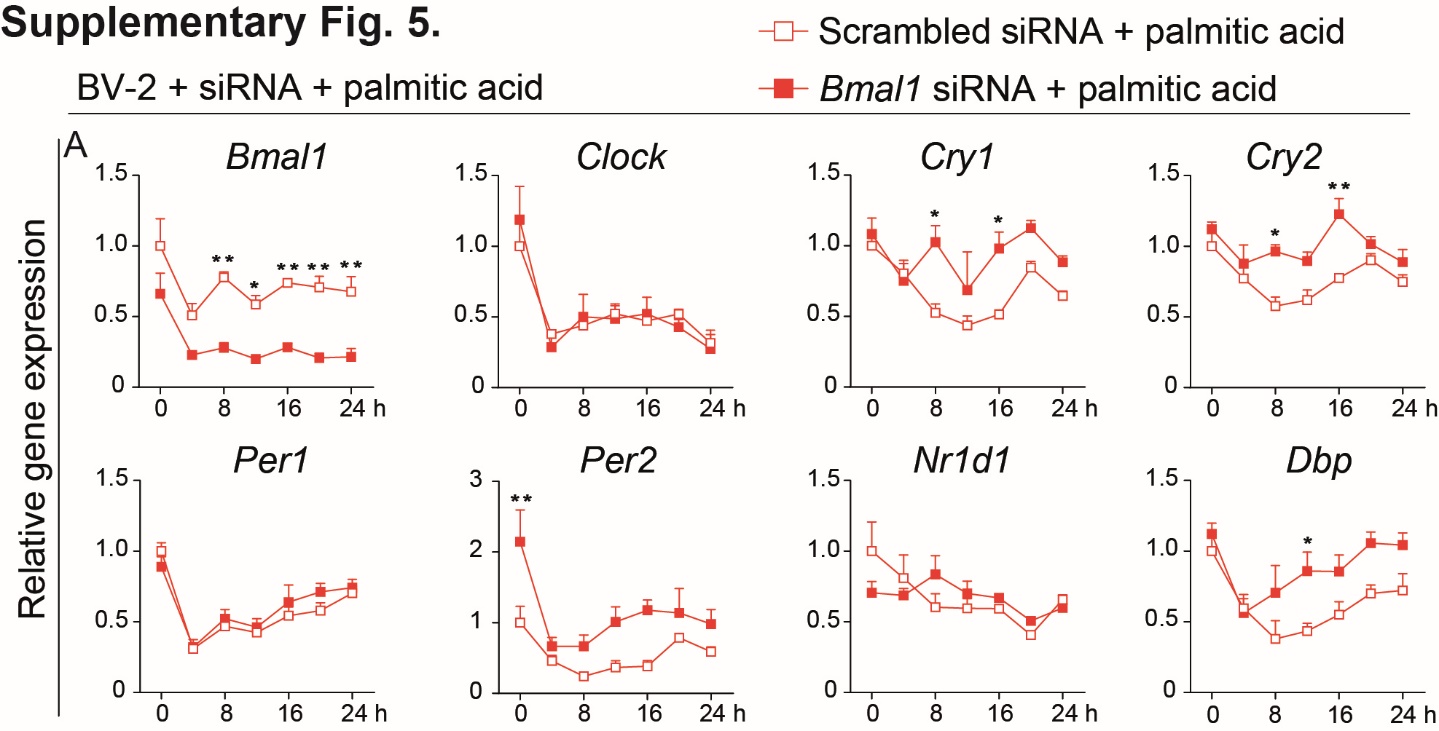


Clock gene expression in scrambled siRNA and *Bmal1* siRNA groups in palmitic acid-treated BV-2 cells. (A) The expression of clock genes was evaluated every 4 h for 24 h in the presence of palmitic acid (n = 3-6 samples per group per time point). Statistical significance was determined using two-way ANOVA. Statistical significance of rhythmic expression was determined by Cosinor analysis. Data are presented as means ± s.e.m. * *P* < 0.05, and ** *P* < 0.01.


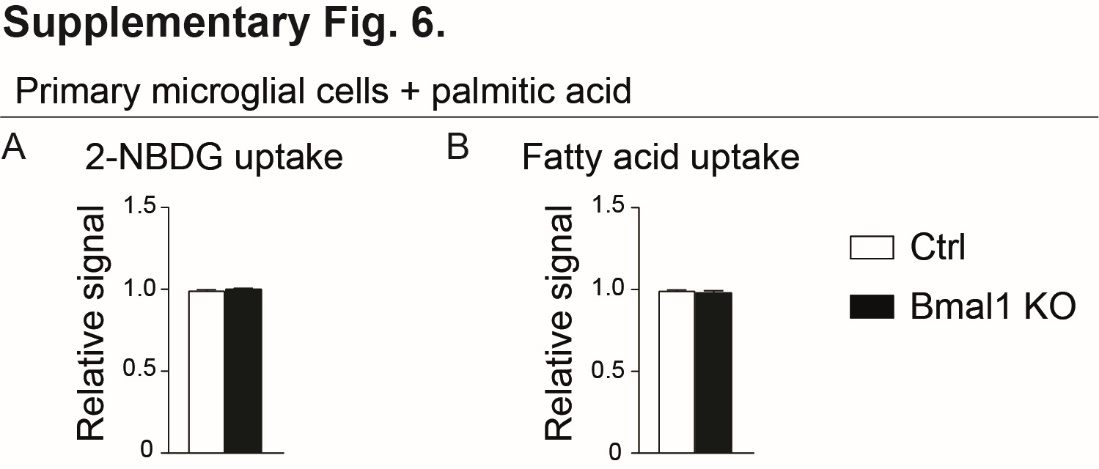


Energy utilization in Ctrl and Bmal1 KO microglial cells treated with palmitic acid. (A-B) 2-NBDG glucose uptake (A), and free fatty acid uptake (B) of Ctrl and Bmal1 KO microglia (n = 5). Statistical significance was determined using two-tailed *t*-test. Data are presented as means ± s.e.m.

**Supplementary Tables**

**Supplementary Table 1. Primers for quantitative PCR**

| Genes | Accession | Forward primer 5’-3’ | Reverse primer 5’-3’ |
| --- | --- | --- | --- |
| *Baml1* | NM_007489 | ACATCACAAGTACGCCTCCC | TGCTGCCTCATCGTTACTGG |
| *Clock* | NM_007715 | CAGCAGTGGATATGGCTTCAGA | GACTGCGGTGTGAGATGACTT |
| *Per1* | NM_011065 | TGTGCATCTGGTAAAGCACCA | TGTACCTGTAGCAAGGAGGCG |
| *Per2* | NM_011066 | TCTTCCAACACTCACCCCAG | CCTCATTAGCCTTCACCTGCTT |
| *Cry1* | NM_007771 | GAGGCACTTACACGTTTGGAA | GCATTCATTCGAGGTCGTTCAA |
| *Cry2* | NM_009963 | TGTGTTCCCAAGGCTGTTCA | TCCCGTTCTTTCCCAAAGGG |
| *Nr1d1* | NM_145434 | ACAGTGATGTTCCTGAGCCG | TTGGTGAAGCGGGAAGTCTC |
| *Dbp* | NM_016974 | TGTGGGAATCTGGATGGCAA | CTAGATGTCAAGCGTGGCGAG |
| *Il1b* | NM_008361 | CAGTTCTGCCATTGACCATC | TCTCACTGAAACTCAGCCGT |
| *Il6* | NM_031168 | GTTCTCTGGGAAATCGTGGA | TGTACTCCAGGTAGCTATGG |
| *Il10* | NM_010548 | ATGCAGGACTTTAAGGGTTACTTG | TAGACACCTTGGTCTTGGAGCTTA |
| *Tnfa* | NM_013693 | TCTCATCAGTTCTATGGCCC | GGGAGTAGACAAGGTACAAC |
| *Glut1* | NM_011400 | TGATGCGGGAGAAGAAGGTC | CCGTGTTGACGATACCGGAG |
| *Glut5* | NM_019741 | TAGCCTGCTTAGTGCTGACG | GATGAGCCCCACAGTGAAGT |
| *Lpl* | NM_008509 | CTCGCTCTCAGATGCCCTAC | AGCAGTTCTCCGATGTCCAC |
| *Pcx* | NM_008797 | AGAGCTGGGTATCCGCACA | CCGCATCTACACCATTTTCCTTG |
| *Gsr* | NM_010344 | CTTGCGTGAATGTTGGATGTG | GCATCCCTTTTCTGCTTGATG |
| *Hmox1* | NM_010442 | ACAGAGGAACACAAAGACCAG | GTGTCTGGGATGAGCTAGTG |
| *Nox2* | NM_007807 | GGGACTGGGCTGTGAATG | TGACCCAAGGAGTTTTCGAG |
| *HPRT1* | NM_013556 | GCAGTACAGCCCCAAAATGG | AACAAAGTCTGGCCTGTATCCAA |

**Supplementary Table 2. One-way ANOVA analyses of daily variation.**

| **Genes** | ***Il1b*** | ***Tnfa*** | ***Il6*** | ***Nox2*** | ***Glut5*** | ***Lpl*** | ***Gsr*** | ***Hmox1*** |
| --- | --- | --- | --- | --- | --- | --- | --- | --- |
| ***P*** | **P<0,0001** | 0,3566 | **0,0475** | 0,7585 | **0,0198** | 0,7228 | **0,0081** | 0,5477 |

Effect of *Time* on gene expression of microglia. *P* < 0.05 (Bold) indicates a significant effect of *Time*.

**Supplementary Table 3. Statistical analyses of gene rhythmicity**

| Genes | Pm | P_A_ | P_B_ | P_C_ | Genes | Pm | P_A_ | P_B_ | P_C_ |
| --- | --- | --- | --- | --- | --- | --- | --- | --- | --- |
| Related to Fig. 1 | | | | | | | | | |
| *Il1b* | P<0,0001 | P<0,0001 | P<0,0001 | 0,0034 | ***Glut5*** | 0,0585 | P<0,0001 | 0,0176 | P<0,0001 |
| *Tnfa* | 0,333 | P<0,0001 | 0,1397 | 0,0005 | ***Lpl*** | 0,3477 | P<0,0001 | 0,1477 | 0,0277 |
| *Il6* | 0,0313 | P<0,0001 | 0,0088 | P<0,0001 | ***Gsr*** | 0,0499 | P<0,0001 | 0,0147 | P<0,0001 |
| *Nox2* | 0.0499 | P<0,0001 | 0.0147 | P<0,0001 | ***Hmox1*** | 0,265 | P<0,0001 | 0,1045 | 0,0996 |
| Related to supplementary Fig. 2 | | | | | | | | | |
| *Il1b* | P<0,0001 | P<0,0001 | P<0,0001 | P<0,0001 | ***Tnfa*** | P<0,0001 | P<0,0001 | P<0,0001 | P<0,0001 |
| *Il6* | 0.0021 | P<0,0001 | 0.0005 | P<0,0001 | ***Il10*** | 0.0210 | P<0,0001 | 0.0059 | P<0,0001 |
| Related to Fig. 3 Scrambled siRNA | | | | | **Related to Fig. 3 *Bmal1* siRNA** | | | | |
| *Bmal1* | 0,0196 | P<0,0001 | 0,0054 | P<0,0001 | ***Bmal1*** | 0,062 | P<0,0001 | 0,0194 | 0,0040 |
| *Clock* | P<0,0001 | P<0,0001 | P<0,0001 | P<0,0001 | ***Clock*** | P<0,0001 | P<0,0001 | P<0,0001 | P<0,0001 |
| *Cry1* | 0,0004 | P<0,0001 | P<0,0001 | P<0,0001 | ***Cry1*** | 0,0001 | P<0,0001 | P<0,0001 | P<0,0001 |
| *Cry2* | 0,1673 | P<0,0001 | 0,0608 | P<0,0001 | ***Cry2*** | 0,1737 | P<0,0001 | 0,0628 | P<0,0001 |
| *Per1* | 0,0135 | P<0,0001 | 0,0036 | P<0,0001 | ***Per1*** | 0,0490 | P<0,0001 | 0,0149 | 0,3705 |
| *Per2* | 0,0539 | P<0,0001 | 0,0165 | 0,0153 | ***Per2*** | 0,1701 | P<0,0001 | 0,0612 | P<0,0001 |
| *Nr1d1* | 0,0002 | P<0,0001 | P<0,0001 | P<0,0001 | ***Nr1d1*** | 0,0007 | P<0,0001 | 0,0002 | P<0,0001 |
| *Dbp* | 0,0109 | P<0,0001 | 0,0028 | 0,6877 | ***Dbp*** | 0,0056 | P<0,0001 | 0,0014 | P<0,0001 |
| *Il1b* | P<0,0001 | P<0,0001 | P<0,0001 | P<0,0001 | ***Il1b*** | 0,0174 | P<0,0001 | 0,0050 | 0,0140 |
| *Tnfa* | P<0,0001 | P<0,0001 | P<0,0001 | P<0,0001 | ***Tnfa*** | P<0,0001 | P<0,0001 | P<0,0001 | P<0,0001 |
| *Il6* | 0.0719 | P<0,0001 | 0.0227 | 0.0002 | ***Il6*** | 0.0127 | P<0,0001 | 0.0033 | P<0,0001 |
| *Il10* | P<0,0001 | P<0,0001 | P<0,0001 | P<0,0001 | ***Il10*** | 0,0013 | P<0,0001 | 0,0003 | P<0,0001 |
| *Glut1* | 0.0607 | P<0,0001 | 0.0191 | 0.0034 | ***Glut1*** | 0.2667 | P<0,0001 | 0.1081 | 0.0649 |
| *Lpl* | 0,1002 | P<0,0001 | 0,0331 | 0,0003 | ***Lpl*** | 0,0005 | P<0,0001 | 0,0001 | P<0,0001 |
| *Pcx* | 0,0564 | P<0,0001 | 0,0179 | P<0,0001 | ***Pcx*** | P<0,0001 | P<0,0001 | P<0,0001 | P<0,0001 |
| *Gsr* | 0,0046 | P<0,0001 | 0,0012 | P<0,0001 | ***Gsr*** | 0,0731 | P<0,0001 | 0,0240 | P<0,0001 |
| *Hmox1* | 0,5464 | P<0,0001 | 0,2759 | 0,0071 | ***Hmox1*** | 0,7167 | P<0,0001 | 0,4178 | P<0,0001 |
| Related to supplementary Fig. 3  Scrambled siRNA + LPS | | | | | **Related to supplementary Fig. 3**  **Bmal1 siRNA + LPS** | | | | |
| *Bmal1* | P<0,0001 | P<0,0001 | P<0,0001 | P<0,0001 | ***Bmal1*** | 0,0010 | P<0,0001 | 0,0003 | P<0,0001 |
| *Clock* | 0,0059 | P<0,0001 | 0,0015 | P<0,0001 | ***Clock*** | 0,0008 | P<0,0001 | 0,0002 | P<0,0001 |
| *Cry1* | 0,1838 | P<0,0001 | 0,0686 | 0,1119 | ***Cry1*** | 0,9104 | P<0,0001 | 0,6688 | 0,2381 |
| *Cry2* | 0,0093 | P<0,0001 | 0,0025 | 0,4610 | ***Cry2*** | 0,5976 | P<0,0001 | 0,3156 | 0,5115 |
| *Per1* | 0,0411 | P<0,0001 | 0,0129 | 0,7308 | ***Per1*** | 0,0034 | P<0,0001 | 0,0009 | 0,9361 |
| *Per2* | 0,0022 | P<0,0001 | 0,0005 | 0,1328 | ***Per2*** | 0,5990 | P<0,0001 | 0,3182 | P<0,0001 |
| *Nr1d1* | 0,5111 | P<0,0001 | 0,2536 | 0,2065 | ***Nr1d1*** | 0,1502 | P<0,0001 | 0,0543 | 0,0001 |
| *Dbp* | 0,1096 | P<0,0001 | 0,0380 | 0,0001 | ***Dbp*** | 0,2204 | P<0,0001 | 0,0863 | P<0,0001 |
| Related to supplementary Fig. 5  Scrambled siRNA + palmitic acid | | | | | **Related to supplementary Fig. 5**  **Bmal1 siRNA + palmitic acid** | | | | |
| *Bmal1* | 0,5230 | P<0,0001 | 0,2649 | P<0,0001 | ***Bmal1*** | 0,3184 | P<0,0001 | 0,1357 | 0,9303 |
| *Clock* | 0,5315 | P<0,0001 | 0,2708 | 0,0006 | ***Clock*** | 0,7186 | P<0,0001 | 0,4255 | 0,6767 |
| *Cry1* | 0,0003 | P<0,0001 | P<0,0001 | 0,8601 | ***Cry1*** | 0,3482 | P<0,0001 | 0,1548 | P<0,0001 |
| *Cry2* | 0,0056 | P<0,0001 | 0,0016 | P<0,0001 | ***Cry2*** | 0,1377 | P<0,0001 | 0,0495 | 0,0022 |
| *Per1* | 0,0007 | P<0,0001 | 0,0002 | P<0,0001 | ***Per1*** | 0,0013 | P<0,0001 | 0,0003 | P<0,0001 |
| *Per2* | 0,0015 | P<0,0001 | 0,0004 | 0,0637 | ***Per2*** | 0,1317 | P<0,0001 | 0,0486 | 0,0001 |
| *Nr1d1* | 0,1017 | P<0,0001 | 0,0372 | 0,0554 | ***Nr1d1*** | 0,0458 | P<0,0001 | 0,0148 | P<0,0001 |
| *Dbp* | 0,0003 | P<0,0001 | P<0,0001 | P<0,0001 | ***Dbp*** | 0,0099 | P<0,0001 | 0,0027 | P<0,0001 |

Data were fitted to the following regression: y = A + B·cos(2π(x−C)/24); A is the mean level; B is the amplitude and C is the acrophase of the fitted rhythm. An overall *p* value (main *p* value, *P*m) was considered to indicate the rhythmicity.
